# Macular Microvasculature Is Different in Patients with Primary Sjögren’s Disease Compared to Healthy Controls

**DOI:** 10.3390/diagnostics15131701

**Published:** 2025-07-03

**Authors:** Gyde Tadsen, Nadine Zehrfeld, Laura Hoffmann, Marten Gehlhaar, Bettina Hohberger, Christian Mardin, Torsten Witte, Carsten Framme, Diana Ernst, Katerina Hufendiek

**Affiliations:** 1 Department of Rheumatology and Immunology, Hannover Medical School, 30625 Hannover, Germany; gyde.tadsen@stud.mh-hannover.de (G.T.); zehrfeld.nadine@mh-hannover.de (N.Z.); witte.torsten@mh-hannover.de (T.W.); 2 Department of Ophthalmology, Hannover Medical School, 30625 Hannover, Germany; laura.hoffmann36@gmail.com (L.H.); marten.gehlhaar@gmail.com (M.G.); framme.carsten@mh-hannover.de (C.F.); 3 Department of Ophthalmology, Friedrich-Alexander-University of Erlangen-Nürnberg, 91054 Erlangen, Germany; bettina.hohberger@uk-erlangen.de (B.H.); christian.mardin@uk-erlangen.de (C.M.)

**Keywords:** Sjögren’s disease, macular microvasculature, OCTA imaging, retinal biomarker

## Abstract

**Background/Objectives**: This study investigates the macular microvasculature in a large cohort of primary Sjögren’s disease (SjD) patients using optical coherence tomography angiography (OCTA), focusing on how disease duration, activity, and hydroxychloroquine (HCQ) treatment influence retinal microcirculation. **Methods:** A total of 106 eyes (53 SjD patients) and 70 eyes (35 age- and gender-matched healthy controls (HCs)) were examined. The vessel area density (VAD, %) and foveal avascular zone (FAZ, mm2) were measured in three retinal layers: Superficial Vascular Plexus (SVP), Intermediate Capillary Plexus (ICP), and Deep Capillary Plexus (DCP), respectively, in three peri-macular circular sectors (c1, c2, c3) each. **Results:** The VAD was significantly lower in c1 of the DCP in SjD compared to HCs (29.14 ± 7.07 vs. 31.78 ± 9.55, *p* = 0.038). The FAZ was significantly larger in SjD in both SVP (0.41 ± 0.13 vs. 0.34, 0.11, *p* < 0.001; Cohen’s |d| = 0.55) and DCP (0.45 ± 0.15 vs. 0.4 ± 0.14, *p* = 0.014; Cohen’s |d| ± 0.38). Significant correlations were observed between the FAZ size and reductions in the VAD in the SVP and DCP (*p* = 0.010, Cohen’s |d| = 0.2; *p* < 0.001, Cohen’s |d| ± 0.26) and across all layers combined (*p* = 0.019, Cohen’s |d| = −0.18). **Conclusions:** There was a negative correlation between the VAD in the DCP and disease duration (ρ = −0.28, *p* = 0.040). No significant correlation was identified between the duration of HCQ intake and the VAD or FAZ. Our findings indicate microvascular alterations in the DCP of SjD, characterized by a reduced VAD and an enlarged FAZ, which may be attributable to inflammatory or arteriosclerotic factors. OCTA may prove to be a valuable tool for the stratification of vascular risk in SjD.

## 1. Introduction

Sjögren’s disease (SjD) is one of the most common connective tissue diseases. The prevalence of SjD is approximately 0.1% to 0.6% of the population worldwide, and the annual incidence is 4/100,000 [1,2,3]. Patients with SjD suffer, in addition to the typical dryness, from early stroke [4] and premature atherosclerosis [5]. Furthermore, cardiovascular risk factors such as arterial hypertension and cholesterinemia are more common in SjD. Its diagnosis is often delayed due to a variable presentation [5,6].

- SjD is characterized by mononuclear cell infiltration of the exocrine glands, particularly the lacrimal glands. This lymphoid infiltration leads to ocular dryness, also known as keratoconjunctivitis sicca. Anterior segment ocular manifestations, such as dry eye, are common in SjD and can have a significant impact on the patients’ quality of life [7]. However, it remains unclear whether or not SjD also affects the retinal microvasculature. Understanding the impact of SjD on the retinal vasculature is important for several reasons. First, changes in the retinal vascular structure may be indicative of inflammatory events in the retinal vasculature in SjD [5,8,9]. A recent review by Hysa et al. [10] highlights how autoimmune rheumatic diseases (ARDs) contribute to ocular microvascular damage through mechanisms such as immune complex formation, complement activation, and antibody-mediated injury to the endothelium. This resulting endothelial dysfunction in the ocular vessels mirrors a broader systemic vascular involvement, suggesting that retinal microvascular alterations may serve as early indicators of widespread vascular damage [10]. Second, these alterations may be linked with other aspects of vascular disease in SjD, such as Raynaud’s syndrome or vasculitis [11,12]. Assessment of the retinal vasculature by optical coherence tomography angiography (OCTA) could potentially predict microvasculature involvement and may help to understand pathophysiological processes in SjD.
- The ocular microvasculature is unique in that it can be directly visualized, imaged, and quantified in a non-invasive manner using OCTA. OCTA detects retinal blood flow down to the capillary level by measuring changes in multiple cross-sectional images (B-frames) of the same area [13,14]. OCTA can be used to measure the size of the foveal avascular zone (FAZ) and the vessel area density (VAD) around the macula [15,16].
- The center of the fovea is called the FAZ. The FAZ is a specialized area of the human macula, nourished by the choroid, which contains the highest density of cone photoreceptors and has the greatest oxygen consumption [17]. The FAZ is highly sensitive to ischemia and may be an indicator of retinal vascular non-perfusion. When the retinal vessels are damaged, the choroid is unable to supply oxygen to the inner layers of the retina [18].
- The retinal microvasculature consists of capillary networks that surround and supply the fovea; the Superficial Vascular Plexus (SVP), the intermediate capillary plexus (ICP), and the Deep Capillary Plexus (DCP). However, these networks remain outside the central foveal avascular zone (FAZ) [19]. The SVP is supplied by the central retinal artery and composed of larger arteries, arterioles, capillaries, venules, and veins primarily in the ganglion cell layer. There are two deeper capillary networks above and below the inner nuclear layer known as the “intermediate” and “deep” capillary plexuses, or ICP and DCP, respectively, which are supplied by vertical anastomoses from the SVP [14]. Changes in capillary density and FAZ size or morphology may reflect changes in retinal perfusion and vascular integrity [20,21].

In view of the paucity of studies on this topic, the aim of this study was twofold: firstly, to compare FAZ and VAD measurements of SjD patients with those of healthy controls (HCs), and secondly, to investigate the effect of several parameters on the VAD/FAZ of SjD patients. The parameters investigated included disease duration (DD), EULAR Sjögren’s syndrome disease activity index (ESSDAI) [22], and hydroxychloroquine (HCQ) intake.

## 2. Materials and Methods

### 2.1. Study Design and Patient Selection

Between September 2022 and June 2023, 166 eyes of 83 consecutive SjD patients visiting the outpatient clinic of the Department of Rheumatology and Immunology at the Hannover Medical School (MHH)—who fulfilled the inclusion criteria and were asked for participation—were examined in the Department of Ophthalmology.

Thirty SjD patients had to be excluded from the study due to poor image quality, increased intraocular pressure, or for eyes that were too myopic or hyperopic. Ocular examinations turned out to be extremely difficult in patients with severe symptoms due to the poor tolerability caused by dry eyes.

HCs were age- and gender-matched in a 2:1 ratio, and 35 (70 eyes) of them were included in the study. Five HCs had to be excluded due to other eye diseases.

### 2.2. Inclusion Criteria

All patients fulfilled the current EULAR/ACR criteria for SjD, had a DD of at least five years, and provided written informed consent [23]. The study was approved by the Ethics Committee of the Hannover Medical School (Ethics Approval No. 8179_BO_S_2018, Amendment).

### 2.3. Exclusion Criteria

**Figure 1:**
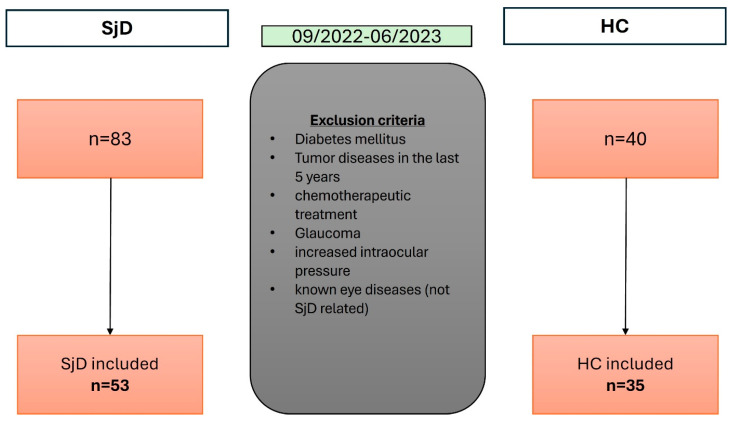
Flowchart of patient recruitment with exclusion criteria consideration.

Participants with additional rheumatic or inflammatory diseases, malignancies treated with chemotherapy in the last five years, diabetes mellitus, glaucoma or elevated intraocular pressure, dense cataract, or retinal and venous alterations such as occlusions and uveitis, were excluded (Figure 1).

To minimize the magnification errors, patients with refractive error > 3.5 diopters were also excluded from the study (*n* = 5).

The symptoms of patients with severe dry eye caused artifacts in spectral domain optical coherence tomography (SD-OCT) and OCTA measurements, resulting in poor image quality and necessitating their exclusion (*n* = 25).

### 2.4. Initial Questionnaire, Clinical Examination, and Cardiovascular Risk Factors

The questionnaire included cardiac risk assessment (LPA, art. hypertension Table 1), assessment of sicca symptoms, and comorbidities. However, questions were also asked about general comparison parameters such as BMI or xerostomia, as well as SjD-related questions such as disease duration month (DDM), antibodies (SS-A and SS-B testing), and treatment medications, particularly HCQ intake.

The ESSDAI, the ESSPRI, and the OSDI scores were also determined. The OSDI is a specific index used to measure ocular dryness and the associated clinical symptoms. It consists of three sections (A, B, C). The index ranges from 0 to 48 with a maximum of 12 points for each section [24]. The subjective symptoms, such as redness, burning, stinging, foreign body sensation, pruritus, and photophobia have been assessed to define dry eye [25]. The disease is characterized as moderately active, as defined by an ESSDAI score of at least five [22].

### 2.5. Methodology of the Ophthalmological Examination

A complete ophthalmic examination, including best corrected visual acuity (BCVA), slit lamp examination, intraocular pressure (IOP) measurement, funduscopic examination, biometry, and SD-OCT, was performed at the first visit. The BCVA was assessed using Snellen charts at five meters [26].

### 2.6. Measurement of the Axial Length

The axial length was measured using swept-source optical biometry (IOLMaster 700, Carl Zeiss meditec AG, 07745 Jena, Germany Softwareversion 1.9038.02, Serialnummer 1242298).

### 2.7. Slit Lamp Examination

The corneal examination with a slit lamp began with an examination of the eyelids for redness, ptosis, and other eyelid abnormalities. The cornea was then examined for signs of dry eye, such as corneal tilt. Fluorescein staining of the cornea was performed for semiquantitative assessment of the corneal surface. In addition, the meibomian glands were examined for blockage, and tear film break-up time was measured by fluorescence staining to assess tear film stability.

### 2.8. Tear Secretion Tests

The examination included the Schirmer I test (without local anesthetic) followed by the Schirmer II test (with local anesthetic). A small absorbent patch was placed in each eye and removed after five minutes [27]. The test was considered positive if the tears flowed down the swab less than five millimeters.

### 2.9. Spectral Domain Optical Coherence Tomography and Optical Coherence Tomography Angiography

SD-OCT and OCTA images were obtained from both eyes using the Heidelberg Spectralis II (Heidelberg Engineering GmbH, Heidelberg, Germany; acquisition software version 6.12.4.0). The SD-OCT device acquired 49 horizontal scans of the fovea. If artifacts were present, manual corrections were made. SD-OCT images were analyzed using the Heidelberg Eye Explorer software (version 2.0, Heidelberg Engineering, Serial number: 07078-S3610, software: Heidelberg, Germany). The software also allows automatic segmentation of specific retinal layers based on predefined algorithms. SD-OCT of the macular and optic nerve was used to exclude any macular disease or glaucomatous changes.

**Figure 2:**
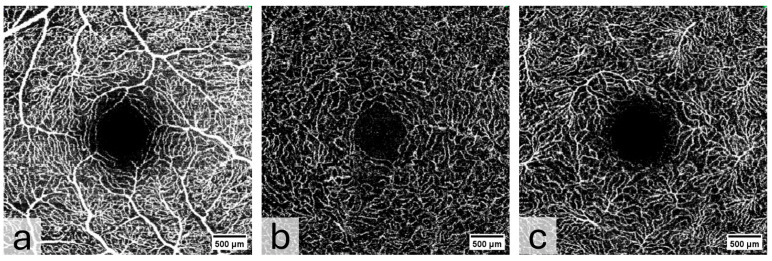
OCTA images of (**a**) Superficial Vascular Plexus (SVP), (**b**) intermediate capillary plexus (ICP), and (**c**) Deep Capillary Plexus (DCP) surrounding the macula. In the middle of the picture is the foveal avascular zone. Illustrations were created with the Heidelberg Eye Explorer.

OCTA was acquired at an angle of 10° × 10° with lateral resolution of 5.85 µm/pixel, resulting in a retinal section of 2.9 × 2.9 mm (total scan size 8.41 mm^2^) (Figure 2). To ensure the correct image interpretation, the quality of the segmentation in horizontal foveal B-scan OCT and en face OCTA maps and in OCTA B-scans was verified by two experienced examiners.

The density of the macular vessels and the FAZ were of particular interest.

### 2.10. Measurement of the Vessel Area Density (VAD, %) and the FAZ (mm^2^)

The obtained scans were exported and analyzed using the Erlangen Angio Tool (EAT) coded in MATLAB (The MathWorks, Inc., Natick, MA, USA, R2017b), as previously described.

The VAD was measured in three layers, the SVP, the ICP, and the DCP, to assess the average value. The SVP was measured from the retinal nerve fiber layer (RNFL) to the inner border of the inner plexiform layer (IPL) and includes the ganglion cell layer. The ICP was measured from the inner border of the IPL to the outer border of the IPL and the DCP, which incorporates the inner nuclear layer and was measured from the outer border of the IPL to the outer plexiform layer (OPL).

**Figure 3:**
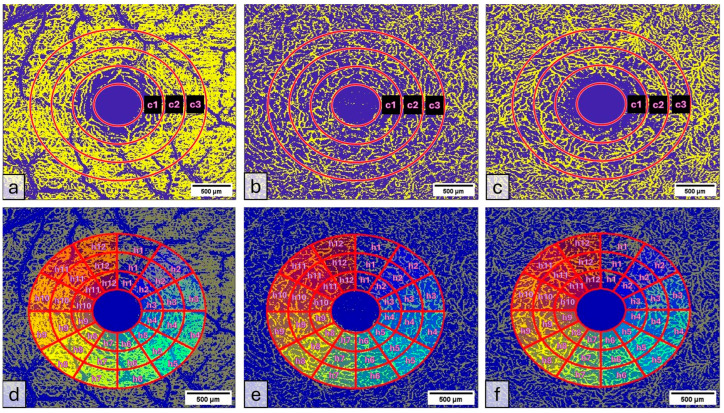
(**a**–**c**): (**a**) Superficial Vascular Plexus (SVP), (**b**) intermediate capillary plexus (ICP), and (**c**) Deep Capillary Plexus (DCP). Region of interest: Macula with circular segmentation of the three peri-macular sectors, c1, c2, c3. The first circle has a radius of 0.40 mm. The radius increment of each circular sector is 0.35. (**d**–**f**): Binarized images analyzed with the Erlangen Angio Tool with circular segmentation and analysis in 12 sectors of (**d**) Superficial Vascular Plexus (SVP), (**e**) intermediate capillary plexus (ICP), and (**f**) Deep Capillary Plexus (DCP) surrounding the macula. In the middle of the picture is the foveal avascular zone.

**Figure 4:**
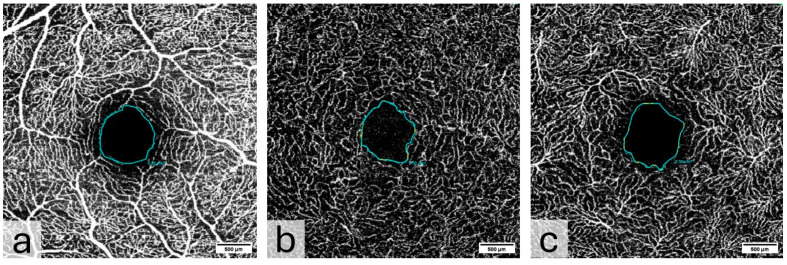
Measurement of the foveal avascular zone (FAZ) in (**a**) Superficial Vascular Plexus (SVP), (**b**) intermediate capillary plexus (ICP), and (**c**) Deep Capillary Plexus (DCP).

The VAD was measured in three circles around the macula (c1, c2, c3, and in full: c1 + c2 + c3) (Figure 3 and Figure 4); the radius of the first circle amounts 0.4 mm. The second circle (c2) radius measures 0.75 mm and the third one (c3) 1.1 mm. Each of these circles was subdivided into 12 smaller areas. For each of these areas, the mean VAD was analyzed using the EAT and calculated in percentage (%).

The FAZ was manually measured in full thickness scans by two different examiners and the mean value of the measurements was noted for statistical evaluation (Figure 4). The total size of the FAZ area (mm^2^) was analyzed and recorded separately for each layer (SVP, ICP, and DCP) of the macula.

All examinations were carried out by trained ophthalmologists, and the findings were independently validated. In the event of differing results, a consensus decision was reached.

### 2.11. Statistical Evaluation

All parameters and results were statistically evaluated using R-Studio (version 4.3.1). As two values are available for each patient (one per eye), we evaluated the VAD and FAZ separately for each eye. For the evaluation of the VAD and FAZ, we treated eyes from the same patients as independent samples. First, descriptive values were calculated, e.g., mean and standard deviation (SD). In addition, skewness and kurtosis were calculated to capture the distributional characteristics.

The Shapiro–Wilk test and histograms were used to test the normal distribution of quantitative data. Two-sample *t*-tests were used for group comparisons, and effect sizes were estimated using Cohen’s d. Pearson’s product–moment correlation coefficients and Spearman’s rank correlation coefficients were used to analyze the associations between two continuous variables. The point–biserial correlation was used to examine the strength and direction of the relationship between HCQ and VAD and FAZ. In all analyses, *p*-values below 0.05 were considered statistically significant [28].

## 3. Results

### 3.1. Patients’ Characteristics Results

A total of 53 SjD patients (106 eyes) and 35 (70 eyes) sex- and age-matched HCs were examined for OCTA analysis. In the SjD group, 41 (77.4%) patients were female, and of the HC group, 27 (77.1%) were female. The exact age and sex distribution is shown in Table 1. In the SjD group, the first diagnosis was made with 8.2 disease duration years (=98.8 disease DDM), and the first date of manifestation was with 12.6 disease duration years (=150.9 DDM) on average.

### 3.2. Initial Questionnaire, Clinical Findings, and Cardiovascular Risk Factors

The mean EULAR Sjögren’s Syndrome Patient Reported Index (ESSPRI) was 6.1, and the ESSDAI was 14.1 (Supplementary Table S1). The mean Ocular Surface Disease Index (OSDI) was 10.1. The distribution of medication scores is shown in Table 1. The Spearman’s rank correlation between the VAD (or FAZ) and the cardiovascular risk factors (LPA) and ESSDAI showed a weak association (Supplementary Table S2). The extended results of the clinical findings and cardiovascular risk factors are presented in Table 1.

### 3.3. Ophthalmological Examination

As would be expected, the BUT and Schirmer test results were found to be lower in patients with SjD when compared to HCs (Supplementary Table S3). No significant differences were observed in either the BCVA or IOP. Furthermore, no significant differences were observed in axial length between patients with SjD and HCs (Supplementary Table S4).

### 3.4. Vessel Area Density (VAD, %)

The mean VAD values were consistently lower in the SjD group than in the HC group (Table 2). The mean VAD in SVP in circle 1 was lower (44.6 ± 10.2 vs. 47.0 ± 10.9, *p*-value = 0.136); in particular, the VAD in DCP in c1 was significantly lower (29.1 ± 7.1 vs. 31.8 ± 9.6, *p* = 0.038). There was also a trend towards reduced DCP in c2 (34.1 ± 7.5 vs. 36.3 ± 7.9 *p* = 0.072) and c3 (32.7 ± 8.1 vs. 34.4 ± 7.8 *p* = 0.180). Slightly reduced VAD in SVP and ICP were not statistically significant.

### 3.5. Foveal Avascular Zone (FAZ, mm^2^)

The FAZ was significantly larger in the SVP (0.41 ± 0.13 vs. 0.34 ± 0.33, *p* < 0.001; Cohen’s |d| = 0.55) and in the DCP (0.45 ± 0.15 vs. 0.4 ± 0.14, *p* = 0.054; Cohen’s |d| = 0.38) in the SjD group compared to HCs (Table 2).

### 3.6. Correlation Analysis Between FAZ and VAD

Pearson’s product–moment correlation analysis showed a moderate negative correlation between the FAZ and VAD in the innermost circle c1 of the SVP (*p* = 0.01; Cohen’s |d| = −0.2) and c1 of the DCP (*p* < 0.001; Cohen’s |d| = −0.26) as well as in full DCP c1 + c2 + c3 (*p* = 0.019; Cohen’s |d| = −0.18 (Supplementary Table S7)).

### 3.7. Correlation Analysis Between VAD/FAZ and SjD Disease Duration (DD)

In the DCP, circle c1 was significantly correlated between the VAD and DD (rho = 0.28, *p* = 0.040). In the SVP and ICP, there was no correlation between disease duration and the VAD (Supplementary Table S5). There was also no significant correlation between the FAZ and DD (Supplementary Table S5).

### 3.8. Comparison Between the Active HCQ Group and the Prior/No HCQ Group and VAD/FAZ

Extended results on correlation analysis between VAD/FAZ and HCQ are presented in Supplementary Table S6. There was no correlation between the intake of HCQ and the VAD or FAZ.

### 3.9. Point–Biserial Correlation of the Relationship Between HCQ Treatment Status and VAD/FAZ

The point–biserial correlation was used to assess the strength and direction of the relationship between HCQ treatment status (active vs. prior/no HCQ as well as active/prior HCQ intake vs. no HCQ intake) and the VAD/FAZ. No statistically significant correlations were observed (Supplementary Table S6).

## 4. Discussion

The results of our study show a difference in the retinal microvasculature in patients with SjD compared to HCs. To our best knowledge, this is the largest cohort of SjD patients being investigated by OCTA that was compared to a sex- and age-matched control group. Patients with SjD show a significantly lower VAD in the innermost circle of the DCP (c1) compared to HCs. This suggests that the deep microvasculature may be more sensitive to vascular changes. In SjD patients, the FAZ was larger in both the SVP and DCP. The VAD was also negatively correlated with disease duration in DCP c1. In a subanalysis, we compared only SjD patients: there was no evidence at the investigation of an association between the duration of HCQ use and the VAD or FAZ. The ESSDAI was also not found to correlate with the FAZ/VAD.

Our findings, including changes in vessel density and morphology, are in line with a few smaller studies of patients with SjD [29,30,31]. Yener et al. (2022) [30] studied 13 female SjD patients (26 eyes) and 20 female HC patients (39 eyes) and found a significantly reduced VAD in the DCP region. No difference in the SVP was found [30]. In another OCTA study by Yang et al. (2022), conjunctival and retinal changes in 12 (24 eyes) SjD patients with 12 (24 eyes) healthy female controls were investigated [29]. They reported a decreased VAD in the SVP and DCP compared to HCs. One reason for the slightly different results of the studies could be due to the small number of patients in their SjD cohort. Wolf et al. (2024) compared patients with SjD and relapsing–remitting multiple sclerosis (RRMS), they found that rarefication of the deep retinal vessels is a unique feature of SjD and is associated with worse visual function compared to RRMS patients [32]. However, a reduction in the VAD was not only found in SjD. Different studies on various connective tissue diseases show similar results to this study: in a study by An et al. (2021) [33] on changes in the VAD and FAZ in systemic lupus erythematode (SLE) patients, a decrease in the VAD was observed in the DCP. In addition, SLE patients showed an enlargement of the FAZ too. This is consistent with our findings in Sjögren’s patients and suggests similar pathophysiological factors [33].

Enlargement of the FAZ due to capillary non-perfusion has previously been reported in diabetic retinopathy or retinal vein occlusion and is often associated with visual loss [34]. We found no significant difference in visual acuity between SjD and HCs (Supplementary Table S3). The negative correlation between the FAZ and VAD in both the SVP and DCP observed in our study aligns with findings from other research, such as An et al. (2021) [33]. We believe that the use of both parameters, FAZ and VAD, together may have a better impact on thse assessment of the retinal microvasculature. Due to the fact that the FAZ is highly sensitive to ischemia [35], we also hypothesize that FAZ alone can be used as a more reliable follow-up or treatment biomarker for cardiovascular risk stratification rather than a diagnostic tool. However, morse longitudinal data is necessary to allow those speculations.

Another reason for changes in the FAZ and VAD could be HCQ. Many SjD patients are treated with HCQ, as it is a very common therapy for arthralgia, morning stiffness, and myalgia, all common symptoms in SjD patients. SD-OCT is one of the screening tools used to diagnose HCQ maculopathy. The retinal toxic effects of HCQ are characterized by thinning of the outer retina and eventual damage to the retinal pigment epithelium. This can lead to loss of vision in advanced stages [36,37]. A study conducted by Ozek et al. (2019) [38] insvestigated the impact of prolonged use of HCQ in patients diagnosed with rheumatoid arthritis [38]. The stsudy revealed a significant reduction in parafoveal, deep temporal, and deep hemi-inferior vascular plexus density among patients who had been administered HCQ for a duration exceeding five years. Nevertheless, the effects of HCQ on the retinal microvasculature in patients with SjD remain unsolved. The research in this area needs to progress, particularly regarding its effects on diseases such as RA, SLE, and SjD, which are associated with systemic inflammation and microvascular changes. Yener et al. (2022) [30] investigated the effect of HCQ therapy on the VAD and FAZ in SjD patients and showed no significant difference in the VAD and FAZ in SjD patients with and without HCQ supplementation [30]. In our study, we also found that there was no significant difference between the use of HCQ and either the FAZ or the VAD. However, the specific effects of HCQ on the VAD are not fully understood, and further research is needed to understand the pathomechanism of microvascular dysfunction. Additionally, the effects of HCQ on vessel density may vary depending on factors such as the underlying condition being treated, the duration and dosage of treatment, and individual patient characteristics that need to be studied in future work.

In another study by Vasilijević et al., examined patients with various autoimmune diseases treated with HCQ showed an increase in FAZ and a decrease in VAD when HCQ was taken for more than five years [39]. This study by Vasilijević et al. found no correlation between DD and OCTA parameters. Both findings are consistent with the results of our study.

In a subanalysis of DD, we found a negative correlation between DD (measured from the date of first manifestation) and VAD reduction in the DCP c1. These findings are also consistent with the results of two other SjD studies by Yener et al. (2022) [30] and Yang et al. [29]. It also supports our hypothesis that changes in the retinal microvasculature as seen in the VAD may depend on the stage of the disease. Therefore, we also suggest that OCTA may be a useful tool for monitoring high-risk SjD patients and stratifying their risk of further retinal damage.

The observed differences in study results may be attributed to the highly variable nature of SjD patient cohorts, particularly when the cohort sizes are limited. SjD is characterized by significant heterogeneity in its manifestationss, with patients exhibiting diverse antibodies or variable organ involvement [40]. Furthermore, the disease’s progression through different stages, in conjunction with the diverse range of treatment modalities employed, may influence the results of OCTA.

Nevertheless, our study has limitations due to its monocentric nature and a selection bias, as 20 SjD patients with severe dryness of the eyes could not tolerate the examinations and had to be excluded due to artifacts or incomplete data, which may have underpowered our results and made it impossible to compare microvascular changes in the subgroup with the greater severity of dry eye disease. The measurement of the VAD and FAZ is dependent on axial length. To mitigate this effect, axial length measurements were performed, and patients with high myopia or hyperopia were excluded from the study.

The FAZ was measured on the prepared images manually by two different experts. A notable strength of this study is the inclusion of the ESSDAI and OSDI in addition to the ESSPRI, which sets it apart from previous studies in this field. In addition to these scores and a detailed questionnaire, the study incorporated several cardiovascular risk factors (LPA, arterial hypertension, etc.) and numerous ocular examinations (Supplementary Table S6). The study revealed a very weak to weak correlation between ESSDAI, LPA, and the FAZ/VAD. This may be attributable to the subclinical nature of these microvascular changes, which do not necessarily correspond to systemic inflammation or organ manifestation. Additionally, it is possible that inclusion of moderate-to-severe Sjögren’s disease patients, who are well controlled and undergoing treatment, may have minimized overt manifestations of the disease, leading to the observed weak association. These findings suggest the need for further research to better understand the relationship between disease activity, treatment, and microvascular changes in Sjögren’s disease.

Our research group, in conjunction with numerous other studies, has demonstrated that patients with SjD exhibit an elevated prevalence of cardiovassascular risk factors, overt cardiovascular disease, and cerebrovascular events [4,41]. In the future, OCTA may have a role in the risk stratification of cardiovascular risk factors and ischemic disease. Nevertheless, further longitudinal studies correlating retinal microvasculature as assessed by OCTA with arteriosclerosis as evaluated by echo-Doppler, in addition to all cardiovascular risk factors and antibody status, may prove useful for a fully comprehensive vascular risk stratification.

## 5. Conclusions

In conclusion, the VAD and FAZ may serve as biomarkers for early detection of microvascular changes in SjD. Evidence of changes in the VAD in DCP c1 and FAZ may indicate inflammatory changes and possible early stages of atherosclerosis of microvasculature in SjD. Longitudinal data would be highly valuable for assessing the progression of microvascular changes in SjD and could enable the investigation of the factors that most influence these changes.

## Figures and Tables

**Table 1:** Baseline demographic data and clinical and cardiovascular risk factors in SjD patients and HCs. Unless otherwise stated, mean and standard deviation are reported.

	Sjögren Disease N = 53	Healthy Controls N = 35
**General data**		
**Mean age [years]**	54.3	55.1
**Female [%]**	77	76
**First diagnosed SjD [mth.]**	98.8 ± 86	
**First manifestation SjD [mth.]**	150.9 ± 97.8	
**Indexes and Tests**		
**ESSPRI** [0–30]	6.1 ± 4.4	0
**ESSDAI-mean**	14.1 ± 1.4	0
**ESSDAI [hematological domain]**	1 ± 0.2	0
**OSDI** [0–48]	10.1 ± 9.1	0
Path. Schirmer I *	23/53 (43.4%)	1/35 (2.9%)
Path. Schirmer II **	26/53 (49.1%)	1/35 (2.9%)
**Path. Salivary gland biopsy**	44/53 (83.0%)	
**Clinical and Cardiovascular risk factors**		
**SSA/Anti-Ro Antibody pos.**	24/53 (45.3%)	0
**SSB/Anti-La Antibody pos.**	5/53 (9.4%)	0
**ANA Antibody pos.**	33/53 (63.2%)	0
**HbA1c [%]**	5.1 ± 0.4	5.2 ± 0.4
**Cholesterol [mmol/L]**	5.± 1.4	5.5 ± 1.2
**HDL-Cholesterol [mmol/L]**	1.8 ± 0.6	1.7 ± 0.5
**LDL-Cholesterol [mmol/L]**	3.1 ± 0.6	3.1 ± 0.6
**Triglyceride [mmol/L]**	1.4 ± 0.9	1.6 ± 1.0
**Lipoprotein A [mmol/L]**	65.9 ± 58.8	38.8 ± 49
**Raynaud’s phenomenon**	19/53	0/35
**Art. hypertension**	16/53 (30%)	8/35 (23%)
**Previous thrombotic events**	8/53	1/35
**Cutaneous vasculitis**	2/53	0/35
**CRP [mg/dL]**	3.0 ± 3.9	2.1 ± 2.2
**BMI**	24.3 ± 5.0	24.3 ± 5.1
**Xerostomy [%]**	64 (34/53)	0 (0/35)
**HCQ therapy**		
**HCQ**	N = 42	
**HCQ act.**	N = 26	
**HCQ duration [mth]**	46	

* without local anesthetic ** with local anesthetic.

**Table 2:** Extended results of VAD and FAZ in Sjögren’s patients compared to healthy controls, *t*-test; values in bold indicate statistical significance (*p* < 0.05). Data are presented as mean ± standard deviation.

Results in Microvasculature			SjD Cohort	HC Cohort	*p* Values
* 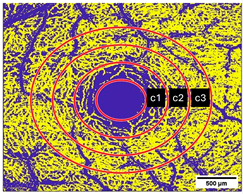 *	**SVP**	VAD c1 [%]	44.6 ± 10.2	47.0 ± 10.9	0.136
		VAD c2 [%]	53.5 ± 10.4	54.0 ± 10.3	0.769
		VAD c3 [%]	47.2 ± 10.1	47.1 ± 10.8	0.955
		VADc1 + c2 + c3 [%]	145.3 ± 28.5	148.4 ± 29.7	0.496
* 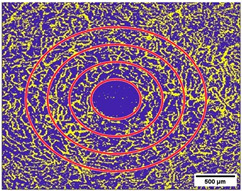 *	**ICP**	VAD c1 [%]	25.8 ± 5.6	26.5 ± 4.7	0.379
		VAD c2 [%]	27.4 ± 6.5	27.7 ± 4.8	0.717
		VAD c3 [%]	26.0 ± 6.8	26.4 ± 5.3	0.636
		VADc1 + c2 + c3 [%]	79.1 ± 18.2	80.8 ± 14.1	0.517
* 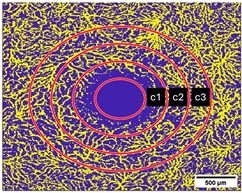 *	**DCP**	VAD c1 [%]	29.1 ± 7.1	31.8 ± 9.6	**0.038**
		VAD c2 [%]	34.1 ± 7.5	36.3 ± 7.9	0.072
		VAD c3 [%]	32.7 ± 8.1	34.4 ± 7.8	0.180
		VAD c1 + c2 + c3 [%]	96.0 ± 21.7	102.4 ± 23.8	0.667
* 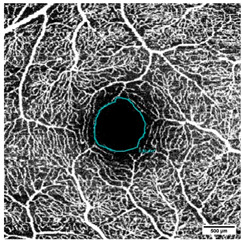 *	**FAZ SVP**	SVP [mm^2^]	0.41 ± 0.13	0.34 ± 0.33	**<0.001,** **Cohen’s d 0.55**
* 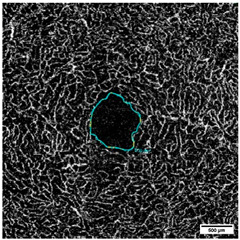 *	**FAZ ICP**	ICP [mm^2^]	0.24 ± 0.10	0.2 ± 0.10	0.082
* 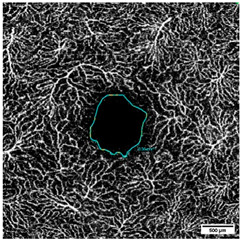 *	**FAZ DCP**	DCP [mm^2^]	0.45 ± 0.15	0.4 ± 0.14	**0.014,** **Cohen’s d 0.38**

## Data Availability

Data can be seen in the online supplement. Additionally, full comprehensive access to all materials is available on reasonable request.

## Supplementary Materials

The following supporting information can be downloaded at: https://www.mdpi.com/article/10.3390/diagnostics15131701/s1, Table S1: The European League Against Rheumatism Sjögren’s Syndrome Disease Activity Index (ESSDAI) was used to assess disease activity. For each time point, the mean ESSDAI score ± standard deviation (SD) is reported to summarize the central tendency and variability within the patient cohort. The relatively high ESSDAI values are explained by the presence of patients with a long disease duration, including cases where Sjögren’s syndrome remained undiagnosed for an extended period. Table S2: Overview of the correlation between the VAD (or FAZ) and the cardiovascular risk factors and ESSDAI (Spearman’s rank correlation) and LPA (Spearman’s rank correlation). Table S3: Overview of various examinations of the anterior chamber of the eye in SjD and HCs for the right eye (oculus dexter (OD)) and the left eye (oculus sinister (OS)): the tear break-up time (BUT) (BUT [seconds] <5 severely reduced, 5–10 reduced, >10 normal). The LIPCOF (lid-parallel conjunctival folds, scale from 10 (no conjunctival fold) to 4 (large conjunctival fold)). The meibomian glands (scale from 0 (atrophy) to 3 (normal, fluid) and provides information about the condition of the secretion of the meibomian glands). The fluorescein staining (Oxford Grading Scale (OGS) from 0 (inconspicuous) to 5 (fluorescence does not wash out of the eye at all too slightly)). Schirmer I and II test in mm. Table S4: Overview of visual acuity, intraocular pressure (IOP), and ocular axial length in SjD and HCs for OD and OS. Table S5: Correlation between DD and the VAD and FAZ (measured in three layers: (SVP, ICP, DCP)). Table S6: Overview of the comparison of VAD and FAZ between the active HCQ intake group and prior/no HCQ group in the three vascular plexuses of the retina (SVP, ICP, DCP), statistically evaluated via R. We used the *t*-test to compare the mean values of the two groups. Overview of the correlation between the VAD (or FAZ) and the intake of HCQ in the three vascular plexuses of the retina (SVP, ICP, DCP), statistically evaluated via R. To examine the association between HCQ intake (column 1: binary variable: intake vs. no intake; column 2: active intake vs. no/prior intake) and retinal vascular density (VAD; continuous variable) or FAZ (continuous variable, a point–biserial correlation was conducted. This method quantifies the linear relationship between a dichotomous and a continuous variable. Prior to the analysis, the VAD values were assessed for approximate normality to meet the assumptions of the test. A significant correlation coefficient would indicate a relationship between medication intake and retinal vascular density. Table S7. Pearson’s product–moment correlation analysis between FAZ and VAD, two-sample *t*-test; values in bold indicate statistical significance (*p* < 0.05).

## Abbreviations

The following abbreviations are used in this manuscript:

ACR/EULAR	American College of Rheumatology/European League Against Rheumatism
BCVA	best corrected visual acuity
BMI	body mass index
BUT	tear break-up time
CRP	C reactive protein
DD	disease duration
DDM	disease duration month
DM	diabetes mellitus
EAT	Erlangen Angio Tool
ESSDAI	EULAR Sjögren’s syndrome disease activity index
ESSPRI	EULAR Sjogren’s Syndrome Patient Reported Index
FAZ	foveal avascular zone
HCs	healthy controls
HCQ	hydroxychloroquine
HDL	high-density lipoprotein
IOP	intraocular pressure
LDL	low-density lipoprotein
LIPCOF	lid-parallel conjunctival folds
LPA	lipoprotein a
OCTA	optical coherence tomography angiography
OD	oculus dexter
OS	oculus sinister
OSDI	Ocular Surface Disease Index
SD-OCT	spectral domain optical coherence tomography
SjD	Sjögren’s disease
SLE	systemic lupus erythematode
SSA/Ro-AB	anti-Sjögren’s syndrome-related antigen A antibodies
SSB/La-AB	anti-Sjögren’s syndrome-related antigen B antibodies
VAD	vessel area density
