# Supplementary material for: Macular Microvasculature Is Different in Patients with Primary Sjögren’s Disease Compared to Healthy Controls
Source: Diagnostics (Basel). 2025 Jul 3;15(13):1701. doi: 10.3390/diagnostics15131701 (PMC12248565; doi:10.3390/diagnostics15131701)
Supplement: Supplementary file 1 [file diagnostics-15-01701-s001.zip › diagnostics-3673501-supplementary.pdf]

Supplementary Data

Suppl. Table S1: *The European League Against Rheumatism Sjögren's Syndrome Disease Activity Index (ESSDAI) was used to assess disease activity. For each time point, the mean ESSDAI score ± standard deviation (SD) is reported to summarize the central tendency and variability within the patient cohort. The relatively high ESSDAI values are explained by the presence of patients with a long disease duration, including cases where Sjögren's syndrome remained undiagnosed for an extended period.*

	**Mean**
**ESSDAI Constitutional**	0.8±0.2
**ESSDAI Lymphadenopathy**	0.4±0.2
**ESSDAI Glandular**	0.5±0.1
**ESSDAI Articular**	1.9±0.2
**ESSDAI Cutaneous**	0.4±0.2
**ESSDAI Pulmonary**	1.2±0.5
**ESSDAI Renal**	0.2±0.2
**ESSDAI Muscular**	1.3±0.4
**ESSDAI PNS**	4.4±0.8
**ESSDAI CNS**	1.0±0.4
**ESSDAI Haematological**	1.0±0.2
**ESSDAI Biological**	0.5±0.1
**ESSDAI Sum**	14.1±1.4

Suppl. Table S2*: Overview of the correlation between the VAD (or FAZ) and the cardiovascular risc factors and ESSDAI (Spearman rank correlation), LPA (Spearman rank correlation)*

	**ESSDAI***	**LPA***
**VAD SVP c1**	OD: -0.17; OS:0.00	OD: -0.12; OS:-0.18
**VAD SVP c2**	OD: -0.12; OS: 0.07	OD: -0.04; OS: -0.17
**VAD SVP c3**	OD: -0.12; OS: 0.07	OD: -0.18; OS: -0.24
**VAD SVP c1+c2+c3**	OD: -0.14; OS: 0.05	OD: -0.15; OS: -0.19
**VAD ICP c1**	OD: -0.16; OS:0.05	OD: -0.04; OS:0.12
**VAD ICP c2**	OD: -0.06; OS: 0.09	OD: -0.05; OS: 0.07
**VAD ICP c3**	OD: 0.02; OS: 0.10	OD: 0.03; OS: -0.01
**VAD ICP c1+c2+c3**	OD: -0.05; OS: 0.09	OD: 0.00; OS: 0.06
**VAD DCP c1**	OD: -0.12; OS:0.00	OD: -0.02; OS: -0.02
**VAD DCP c2**	OD: -0.06; OS:0.13	OD: -0.12; OS: -0.01
**VAD DCP c3**	OD: -0.01; OS: 0.06	OD: -0.16; OS: 0.03
**VAD DCP c1+c2+c3**	OD: -0.09; OS:0.07	OD: -0.13; OS: 0.00
**FAZ SVP**	OD: 0.18; OS: 0.06	OD: 0.07; OS: 0.06
**FAZ ICP**	OD: 0.20; OS: 0.32	OD: 0.05; OS: 0.01
**FAZ DCP**	OD: .11; OS: 0.12	OD: -0.04; OS: 0.11

Suppl. Table S3*: Overview of various examinations of the anterior chamber of the eye in SjD and HC for the right eye (oculus dexter (OD)) and the left eye (oculus sinister (OS)): The tear break up time (BUT) (BUT [seconds] <5=severely reduced, 5-10= reduced, >10 normal). The LIPCOF (lid-parallel conjunctival folds, scale from 10 (no conjunctival fold) to 4 (large conjunctival fold). The meibomian glands (scale from 0 (atrophy) to 3 (normal, fluid) and provides information about the condition of the secretion of the meibomian glands. The fluorescein staining (Oxford Grading Scale (OGS) from 0 (inconspicuous) to 5 (fluorescence does not wash out of the eye at all too slightly). Schirmer I and II Test in mm.*

	**SjD OD**	**SjD OS**	**SjD**	**HC OD**	**HC OS**	**HC**
**BUT [sec.]**	5.44±2.91	5.19±2.3	5.32±2.97	15.4±2.96	16.8±4.87	16.1±4.54
**LIPCOF [scale]**	1.43±0.81	1.32±0.65	1.37±0.71	0.62±0.63	0.7±0.54	066±0.54
**Meibom Glands [scale]**	2.67±0.58	2.75±0.55	2.68±0.55	3±0.24	3±0.35	3.06±0.24
**Fluorescein (OGS)**	1.66±0.98	1.78±0.95	1.66±0.94	0.86±0.84	0.88±0.94	0.87±0.83
**Schirmer I [mm]**	7.65±9.1	7.29±9.12	7.47±8.78	18.6±7.89	19.32±7.81	18.97±7.5
**Schirmer II [mm]**	4.75±6.1	4.36±6.52	4.5±6.14	12.64±7.14	12.98±6.81	12.81±6.77

Suppl. Table S4: *Overview of visual acuity, intraocular pressure (IOP) and ocular axial length in SjD and HC for OD and OS*

	**SjD OD**	**SjD OS**	**HC OD**	**HC OS**
**Visual acuity**	1.08±0.18	1.03±0.21	1.02±0.21	1.06±0.19
**IOP**	17.36±3.72	17.56±4.31	15.38±2.59	15.69±2.53
**Ocular axial length**	23.7±1.04	23.43±2.83	24.6±1.41	24.54±1.37

Suppl. Table S5*: Correlation between DD and the VAD and FAZ (measured in three layers: (SVP, ICP, DCP).*

	**Spearman rho**	**p-value**
**SVP c1**	-0.07	0.623
**SVP c2**	-0.07	0.638
**SVP c3**	-0.05	0.733
**SVP c1, c2, c3**	-0.07	0.594
**ICP c1**	-0.03	0.805
**ICP c2**	-0.08	0.567
**ICP c3**	-0.08	0.559
**ICP c1, c2, c3**	-0.06	0.664
**DCP c1**	**-0.28**	**0.040**
**DCP c2**	-0.23	0.091
**DCP c3**	-0.16	0.251

**DCP c1, c2, c3**	-0.25	0.067
**FAZ SVP**	-0.102	0.474
**FAZ ICP**	-0.135	0.346
**FAZ DCP**	-0.14	0.333

***Suppl. Table S6.**: Overview in row 2-4 of the comparison of VAD and FAZ between the active HCQ intake group an prior/no HCQ group in the three vascular plexus of the retina (SVP, ICP, DCP) statistically evaluated via R. We used the t-test to compare the mean values of the two groups*

Overview in row 5 and 6 of the correlation between the VAD (or FAZ) and the intake of HCQ in the three vascular plexus of the retina (SVP, ICP, DCP) statistically evaluated via R. To examine the association between HCQ intake (column 5: binary variable: intake vs. no intake and column 6 active intake vs. no/prior intake) and retinal vascular density (VAD; continuous variable) or FAZ (continuous variable, a point-biserial correlation was conducted. This method quantifies the linear relationship between a dichotomous and a continuous variable. Prior to the analysis, the VAD values were assessed for approximate normality to meet the assumptions of the test. A significant correlation coefficient would indicate a relationship between medication intake and retinal vascular density

	**Active HCQ intake (N=26) Mean value**	**prior/no HCQ intake (N=27) Mean value**	**p-value**	**Correlation no HCQ intake vs. prior /active HCQ intake**	**Correlation prior/no HCQ intake vs. active HCQ intake**
**VAD HCQ SVP c1**	45.3	43.5	0.508	-0.2	0.1
**VAD HCQ SVP c2**	54.4	52.4	0.425	-0.2	0.1
**VAD HCQ SVP c3**	47.5	46.7	0.748	-0.1	0.04
**VAD HCQ SVP c1+c2+c3**	147.2	142.6	0.514	-0.2	0.1
**VAD HCQ ICP c1**	26.5	24.9	0.236	0.02	0.2
**VAD HCQ ICP c2**	28.4	26.2	0.144	0.04	0.2
**VAD HCQ ICP c3**	26.9	25	0.196	0.07	0.18
**VAD HCQ ICP c1+c2+c3**	81.8	76.1	0.170	0.05	0.19
**VAD HCQ DCP c1**	29.7	28.5	0.479	-0.1	0.1
**VAD HCQ DCP c2**	34.6	33.6	0.585	-0.1	0.08
**VAD HCQ DCP c3**	33.4	32.1	0.475	-0.09	0.1
**VAD HCQ DCP c1+c2+c3**	97.6	94.1	0.475	-0.11	0.1
**FAZ HCQ SVP**	0.41	0.42	0.839	-0.1	-0.03
**FAZ HCQ ICP**	0.24	0.24	0.818	-0.1	-0.03
**FAZ HCQ DCP**	0.46	0.45	0.854	0.1	0.03

Suppl. Table S7. *Pearson’s product-moment correlation analysis between FAZ and VAD, values in bold indicate statistical significance (p<0.05).*

	**p-value**	**Cohens d**
**SVP c1**	**0.010**	**-0.2**
**SVP c2**		0.1
**SVP c3**		0.15
**SVP c1+c2+c3**		0.02
**ICP c1**		0.07
**ICP c2**		0.13
**ICP c3**		0.11
**ICP c1+c2+c3**		0.12
**DCP c1**	**<0.001**	**-0.26**
**DCP c2**		-0.13
**DCP c3**		-0.11
**DCP c1+c2+c3**	**0.019**	**-0.18**
